# Foliar P Application Cannot Fully Restore Photosynthetic Capacity, P Nutrient Status, and Growth of P Deficient Maize (*Zea mays* L.)

**DOI:** 10.3390/plants11212986

**Published:** 2022-11-05

**Authors:** Jon Niklas Henningsen, Bruno Maximilian Görlach, Victoria Fernández, Jasper Lauritz Dölger, Andreas Buhk, Karl Hermann Mühling

**Affiliations:** 1 Institute of Plant Nutrition and Soil Science, Kiel University, 24118 Kiel, Germany; 2 German Agricultural Society E.V., 60489 Frankfurt am Main, Germany; 3 Systems and Natural Resources Department, School of Forest Engineering, Technical University of Madrid, Ciudad Universitaria S/N, 28040 Madrid, Spain

**Keywords:** chlorophyll, foliar absorption, foliar sprays, maize, phosphorus deficiency, phosphorus translocation, photosynthesis

## Abstract

The essential plant nutrient phosphorus (P) is key for numerous structures and processes in crops and its deficiency can severely restrict yield and quality. As soil P availability for plant uptake is often limited, foliar P application can be an alternative means of supplying P to the plants during the growth period. This study was aimed at investigating the effect of foliar P application on photosynthetic parameters, P nutritional status, and growth of P deficient maize over time. Plants of *Zea mays* L. cv. Keops were grown with deficient and sufficient amounts of P in hydroponics. Foliar P treatments were applied to P deficient plants and several physiological parameters were monitored for 21 days. The variables measured were leaf gas exchange parameters, SPAD values, foliar P absorption, re-translocation rates, and plant biomass production. Foliar P application significantly increased CO_2_-assimilation and SPAD values and additionally enhanced biomass production in all plant components. Elemental analysis revealed increased tissue P concentrations following foliar P application compared to P deficient plants. While increased growth of P-deficient plants was steadily promoted by foliar P spraying for the entire experimental period, the positive effect on CO_2_ assimilation and P concentration was transient and vanished some days after the foliar treatment. P deficiency markedly impaired the efficiency of physiological processes of maize plants. As a conclusion, foliar P fertilisation improved physiological and agronomical plant parameters over time, but failed to restore plant functionality of P deficient maize plants during a prolonged experimental period.

## 1. Introduction

Phosphorus is one of the most important and limiting nutrients for the growth of highly productive staple crops [1,2]. According to MacDonald et al. [3], about 30% of the world’s arable soils are deficient in P. For this reason, commercial crops are commonly supplemented with P soil fertilisation [4]. However, even with sufficient P supply, the availability of P for plants is often limited, which is mainly due to the high sorption capacity of inorganic P (P_i_) towards iron, aluminium, and calcium cations, as well as to the surface of soil particles [5,6]. As a consequence, only approximately 20% of P fertiliser added to the soil is taken up and potentially utilized by the plant in the year of application [7]. The resulting accumulation of P in the soil increases the risk of diffuse losses [8,9]. In particular, the loss of P via erosion and runoff into surface waters can have lasting negative effects on aquatic ecosystems through eutrophication [10,11]. This problem could be further exacerbated in the future by increasing extreme weather events, such as heavy rainfall [12,13]. The change in the global climate will also lead to longer and more intensive periods of drought [14,15]. Hence, there will be a reduced diffusion of P into the rhizosphere and thus a lower uptake of P by plants [16]. Besides, or even due to its functions in energy transfer and carbon partitioning and as a component of nucleic acids as well as biomembranes, P is a key element for photosynthesis [17]. As a consequence of P deficiency, essential steps of photosynthesis such as the regeneration of RuBP in the Calvin cycle [18], synthesis of ATP [19] or the electron transport chain between PSII and PSI [20,21] are disturbed and photosynthetic performance is though reduced [22].

In the present investigation, maize (*Zea mays* L.) is used, as it is the second most harvested crop in the world and, together with wheat and rice, provides 30% of the calorie needs of at least 4.5 billion people [23]. Furthermore, maize plants are particularly susceptible to P deficiency during youth development and therefore have a high P fertiliser requirement at the beginning of vegetation [24]. Often, a P application at sowing can provide sufficient P for the entire growing season. However, if the P demand of the plants is higher (e.g., favourable growth conditions) or the P availability in the soil is lower (e.g., drought) than predicted, additional in-season soil fertilisation can no longer be applied efficiently and without risking damage to the plants [25]. For this reason, P foliar application has become increasingly popular in recent years as a way of supplying P during crop growth and increasing the P use efficiency. However, while foliar fertilisation in horticultural crops is a common management tool with a known impact, numerous studies have been conducted on the use of foliar fertilisation in arable crops, but with varying results in terms of effectiveness [25,26,27,28,29]. Even though Noack et al. [30] consider foliar fertilisation as a viable possibility for arable crops, there is still a major lack of knowledge to fully understand the underlying mechanisms [31,32].

Foliar sprayed agrochemicals first interact with the aerial plant surface, which is almost fully covered with a cuticle [33]. This specialized part of the primary cell wall consists of nano-scale structured hydrophilic (polysaccharides) and hydrophobic (mainly waxes and cutin) areas, often covered with epicuticular waxes, which exert a barrier function. Another main factor for topographical heterogeneity of plant surfaces is the micro-scale roughness, which is determined by the presence of e.g., trichomes and stomata [31,34,35]. The micro- and nano-scale roughness have a significant influence on the interaction between the sprayed agrochemical drop and plant surfaces and thus initially on adherence or repellence and consequently also on the potential uptake of the foliar fertiliser [31,36,37]. Strictly speaking, the uptake of solutes like foliar fertiliser sprays is at first not an active mechanism, but rather a passive diffusion process, of which many underlying mechanisms are still unclear [31]. Since one of the chemical constituents of the cuticle is polysaccharides, water sorption to these polar functional groups can alter the volume of the cuticle. It is hypothesized that transport mechanisms are associated with this process of swelling and shrinking [38,39,40]. An important pathway for the uptake of foliar-applied nutrient ions, such as P, is open stomata [41]. The guard cells form a slim water film on the surface, along which solutes can diffuse into the mesophyll [42,43]. Penetration of nutrients also takes place via trichomes. In recent years, this has been demonstrated in particular for Zn, using synchrotron-based X-ray fluorescence microscopy on soybean (*Glycine max*) and sunflower (*Helianthus annuus*) [44,45,46]. Uptake was particularly apparent via trichomes located on top of bundle sheath extensions [44,47].

Foliar P application in dryland cereal crops has been investigated in several early studies, as reviewed by Noack et al. [30]. In particular, the effects on biomass formation, adequate timing, as well as the optimal concentration of foliar P application were the focus of investigations. In recent years, some foliar P application studies were carried out with P deficient plant species such as wheat [25,48,49,50,51,52], barley [20,21,53,54,55] or maize [28,56,57]. It was shown that P deficiency can lead to alterations in the composition of plant tissues and epidermal structures such as stomata and trichomes, which ultimately affects the rate of uptake of P via foliage. The effect of P deficiency is thereby strongly dependent on the type of crop, among other factors. While for wheat, the number of stomata and trichomes was strongly reduced and P foliar absorption no longer takes place [25], for barley mainly the tissue structure (cuticle, epidermal cell wall, polysaccharides) was changed and P continued to be absorbed, albeit at a reduced rate which could not restore functionality [53]. Foliar P application in P deficient maize plants led to an increase in P concentration and a short-term increase in biomass [28,56,57]. Using high-resolution bioimaging techniques, Arsic et al. [53,54] recently showed that the uptake of P was mainly associated with trichomes and fiber cells, while P was subsequently translocated via bundle sheath extensions.

Recent studies on foliar P fertilisation in maize shed light on key agronomical parameters as well as on translocation processes [28,56,57]. Nevertheless, there are still many knowledge gaps for gaining a complete understanding of the absorption, translocation, and influence on plant physiological processes of foliar-applied P, especially within longer experimental setups. Hence, a study was carried out with P deficient and sufficient maize plants, in which the effect of foliar P resupply was mainly assessed in terms of the rate of P absorption and transport in different plant components, gas exchange parameters, and biomass production over an experimental timespan of 14 and 21 days. In this investigation, we aimed to answer the following questions: (1) how long do the increased tissue P concentrations measured shortly after P foliar spraying of P-deficient maize plants last in time? (2) which plant components have the highest exogenous P accumulation rates following foliar P application? (3) does foliar P application improve gas exchange parameters of P deficient plants? (4) how many days after P foliar supply of P-deficient maize plants is biomass formation impaired?

## 2. Results

### 2.1. P Concentration in Distinct Plant Components after Foliar P Application

**Figure 1:**
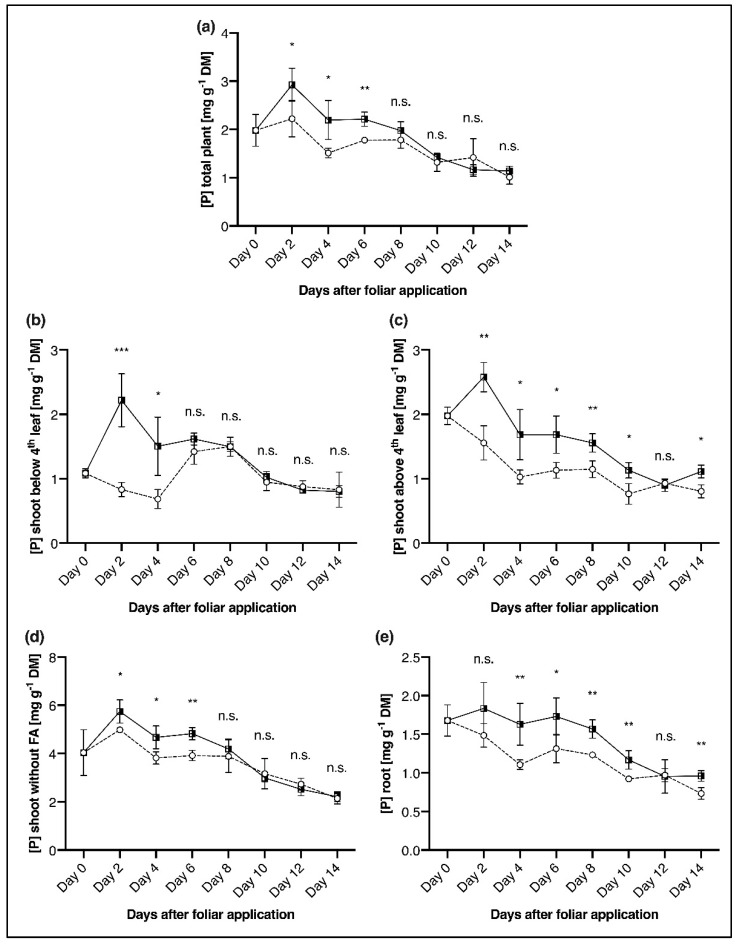
Total phosphorus concentration [P] of different maize plant tissues, analysed over a period of 14 days after foliar P spraying (applied at day 0). Comparison of P deficient control plants (P_def_-control, 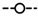) and P deficient plants sprayed with P (P_def_ + FA, 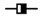). (**a**) total plant, (**b**) shoot below the 4th leaf (4th leaf was the youngest fully emerged leaf), (**c**) shoot above the 4th leaf, (**d**) shoot without foliar application—leaves that were still rolled up and encased by other leaves at the time of foliar application and thus did not receive direct contact with the foliar fertiliser, (**e**) root. Datapoints represent means + standard deviation (*n* = 4). * = *p* ≤ 0.05; ** = *p* ≤ 0.01; *** = *p* ≤ 0.001; n.s. = not significant (*t*-Test).

Foliar P application significantly increased the P concentration in the total plant as well as in all differentiated plant components over time compared to the P-deficient control plants. The maximum P concentration of the whole measuring period was determined already two days after foliar spraying (Figure 1). At a whole plant level, significant differences between the variants, following foliar application were detectable up to the 6th day, with values dropping to the level of P deficient plants thereafter (Figure 1a). A similar pattern was recorded for the shoot below the 4th leaf, but here P concentrations significantly increased only up to the 4th day. In this plant component, the difference in P concentration was the most significant of all five components with P concentrations of 0.83 in P deficient control plants and 2.22 mg P g^−1^ dry matter (DM) in plants with foliar application on day 2, respectively (Figure 1b). In the shoot above the 4th leave and the root, P concentration was increased the longest. After P foliar application, it was higher by 1.5-fold (Ø 0.60 mg P g^−1^ DM) in the shoot above the 4th leave and by 1.3-fold (Ø 0.37 mg P g^−1^ DM) in the root over a period of eight days (Figure 1c,e). The highest P concentration from all plant components was examined in the shoot without foliar application. Nevertheless, the difference in P concentration between the variants was the smallest in this component. Phosphorus concentrations were significantly lower in the P deficient control plants from the 2nd to the 6th day, compared to foliar P treated plants. However, from the 8th day onwards, the two variants converged again, and overall, tissue P concentrations decreased from 4.99 to 2.14 mg P g^−1^ DM in P deficient control plants and from 5.74 to 2.21 mg P g^−1^ DM in plants sprayed with P (Figure 1d). From these results, it is derived that foliar P application led to significantly higher P concentrations in all plant components, which however decreased over time to values within the range of P deficient plants. The intensity and duration of this increase in P concentration associated with foliar P spraying depended on the particular plant component, with higher tissue *p* values being determined in sink organs, such as roots and younger leaves (Figure 1).

### 2.2. Plant Growth

**Figure 2:**
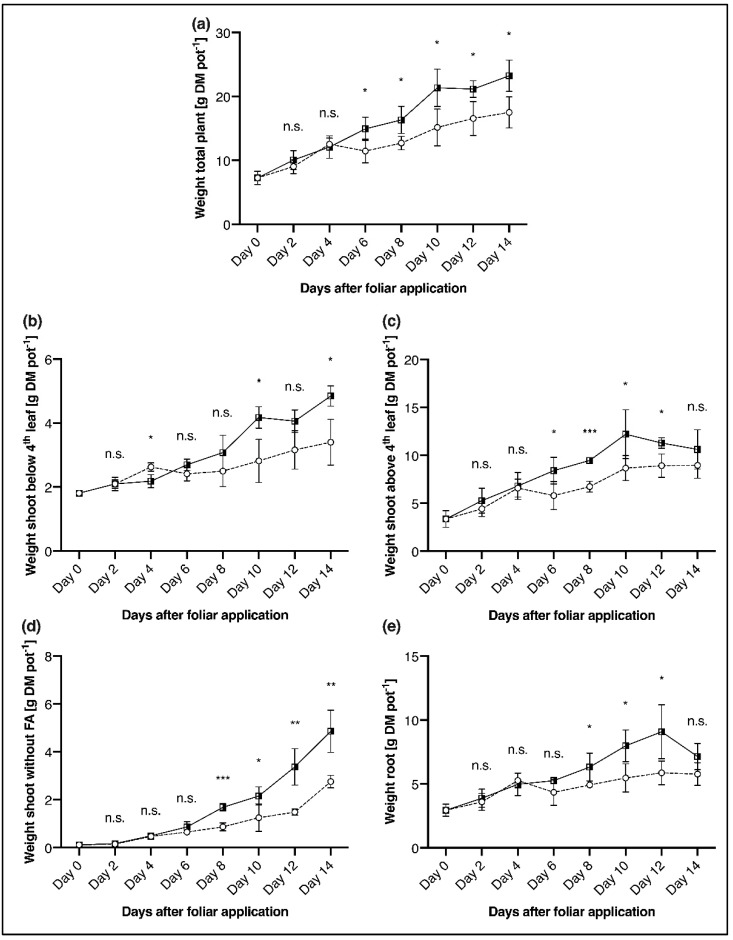
Dry matter (DM) of different maize plant tissues, analysed over a period of 14 days after foliar P spraying (applied at day 0). Comparison of P deficient control plants (P_def_-control, 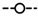) and P deficient plants sprayed with P (P_def_ + FA, 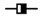). (**a**) total plant, (**b**) shoot below the 4th leaf (4th leaf was the youngest fully emerged leaf), (**c**) shoot above the 4th leaf, (**d**) shoot without foliar application—leaves that were still rolled up and encased by other leaves at the time of foliar application and thus did not receive direct contact with the foliar fertiliser, (**e**) root. Datapoints represent means + standard deviation (*n* = 4). * = *p* ≤ 0.05; ** = *p* ≤ 0.01; *** = *p* ≤ 0.001; n.s. = not significant (*t*-Test).

**Figure 3:**
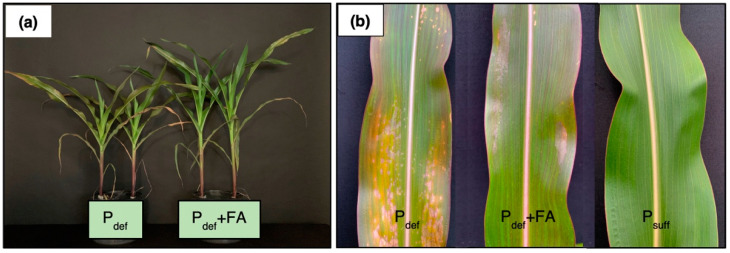
Phenological appearance of maize plants due to their phosphorus nutritional status. (**a**) Comparison of P deficient control plants (P_def_) with P deficient plants sprayed with P (P_def_ + FA). (**b**) Comparison of the 5th leaf of P deficient control plants (P_def_) with P deficient plants sprayed with P (P_def_ + FA) and with P sufficient control plants (P_suff_). Representative plants were selected 14 days after the phosphorus foliar application.

At the end of the 14-day measuring period, 25% higher dry matter (DM) was formed as a result of foliar P application compared to non-sprayed, P deficient plants (Figure 2a and Figure 3a). Already, from the 6th day after foliar application, a significantly higher DM was determined in the total plant (Figure 2a). This response in DM to foliar application was also apparent in all the distinct plant components (Figure 2b–e).

Nevertheless, the intensity and timing of the biomass increase were different depending on the component. Thus, the increase in DM for the foliar applied plants from day 0 to day 14 was greatest in the shoot without foliar P application (41-fold, Figure 2d), while it was getting smaller in the shoot above the 4th leaf (2.2-fold, Figure 2c) in the shoot below 4th leaf (1.7-fold, Figure 2b), and the root (1.4-fold, Figure 2e). A similar development is also evident when examining the differences in DM on day 14 between the P deficient control plants and the plants with P foliar application. So, in the P foliar applied plants, the DM in the shoot without foliar application is 43% higher (Figure 2d), while it is clearly lower in the shoot below the 4th leaf (30%, Figure 2b), in the root (19%, Figure 2e) and the shoot above the 4th leaf (16%, Figure 2c). While in the whole plant and the shoot above 4th leaf a significantly higher DM formation after P foliar application was evident from day 6, this point in time differs slightly for the differentiated plant components. For the shoot without foliar application and the root, this significant change happened on day 8, and for the shoot below the 4th leaf only on day 10 (Figure 2). Besides restricted plant growth, early senescence was observed in the P deficient plants, which could be delayed by P foliar application (Figure 3b).

### 2.3. Net Photosynthesis and Transpiration

**Figure 4:**
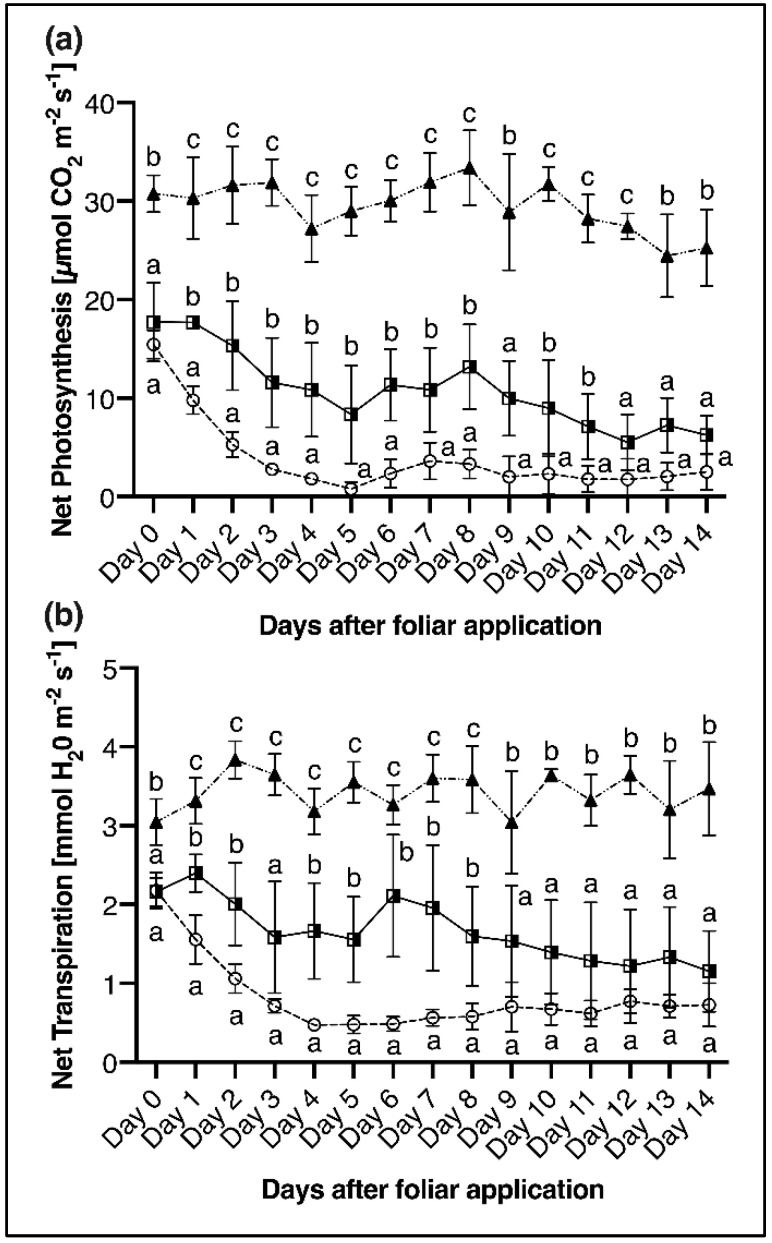
(**a**) Net photosynthesis and (**b**) net transpiration, measured on the youngest fully emerged leaf (4th leaf) of maize plants over a period of 14 days after foliar P spraying (applied at day 0). Treatments correspond to the following symbols: P deficient control plants (P_def_-control, 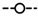), P deficient plants sprayed with P (P_def_ + FA, 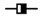) and P sufficient control plants (P_suff_-control, 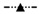). Datapoints represent means + standard deviation (*n* = 3). Letters indicate significant differences between treatments (ANOVA with Duncan test, *p* ≤ 0.05).

**Figure 5:**
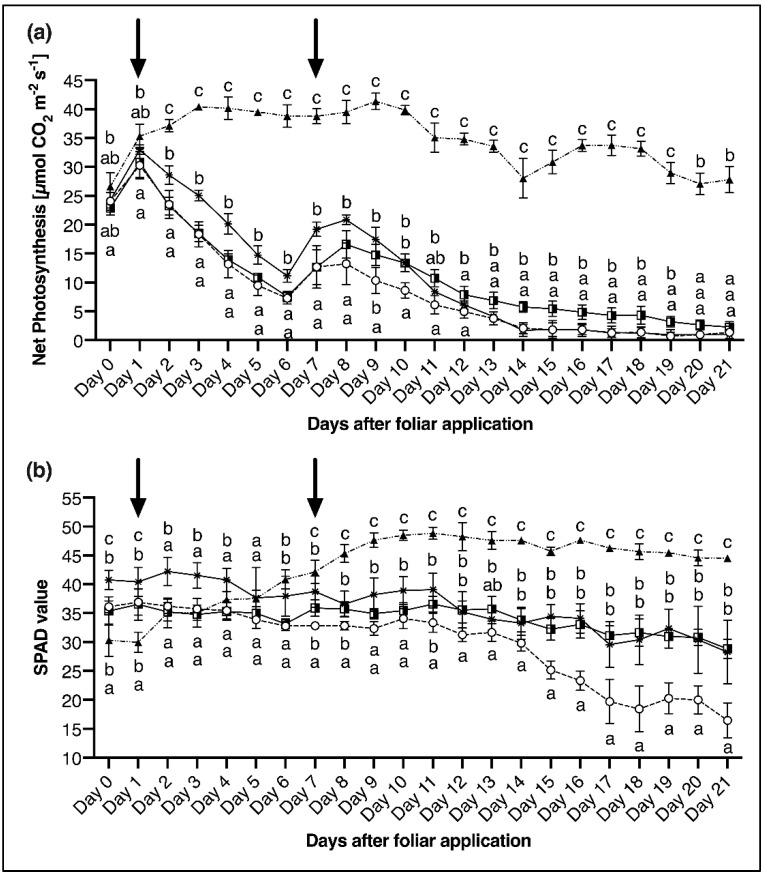
(**a**) Net photosynthesis and (**b**) SPAD values, measured on the 4th leaf of maize plants over a period of 21 days. Two phosphorus foliar sprays were applied on day 1 and day 7. Treatments correspond to the following symbols: P deficient control plants (P_def_-control, 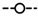), P deficient plants sprayed with P on day 1 (P_def_ + FA1, 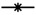), P deficient plants sprayed with P on day 7 (P_def_ + FA2, 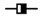), P sufficient control plants (P_suff_-control, 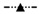). Black arrows (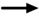) indicate the dates of P foliar application. Datapoints represent means + standard deviation (*n* = 4). Letters indicate significant differences between treatments (ANOVA with Duncan test, *p* ≤ 0.05).

Gas exchange measurements have revealed significant differences in net photosynthetic and net transpiration rates between P deficient and P sufficient plants (Figure 4 and Figure 5a). The rate of CO_2_ assimilation in the 4th leaf of P sufficient plants was subjected to minor fluctuations during the measuring period but remained around 30 to 40 µmol CO_2_ m^−2^ s^−1^. In the P deficient plants, however, the assimilation rate initially decreased within one week from 15 to about 2 µmol CO_2_ m^−2^ s^−1^ in the 1st experiment (30 DAS, Figure 4a) and from 24 to about 7 µmol CO_2_ m^−2^ s^−1^ in the 2nd experiment (23 DAS, Figure 5a). Subsequently, the CO_2_ assimilation rate remained at a steady low level in experiment 1, while it temporarily increases in the course of experiment 2, which is associated with the change of the nutrient solution on day 6 and the supply of a small amount of KH_2_PO_4_ (Figure 4 and Figure 5a). The transpiration rate followed a similar trend as the photosynthesis rate and was significantly lower in P deficient than in the P sufficient control plants at any time during the measuring period (Figure 4b). A resupply of P to deficient plants via foliar P application led to a temporarily significantly reduced decline in CO_2_ assimilation and transpiration rates compared to P deficient control plants shortly after foliar application. In the last 3rd of the measuring period on day 12 (photosynthesis rate) and day 9 (transpiration rate), the values of the P deficient control and the P foliar applied plants approached again and remained on a common, constantly low level. Nevertheless, the plants that received the foliar P treatment had an average of 2.8-fold higher photosynthesis and 1.2-fold higher transpiration over the entire measuring period, compared to the plants without additional P fertilisation (Figure 4).

In the 2nd experiment, foliar applications were carried out at two different times (24, 30 DAS/ Day 1 and 7 in the measuring period) on two different groups of plants. By starting the measurement earlier and on younger plants than in the 1st experiment, it could be examined that P deficient and P sufficient plants show the same course of CO_2_ assimilation until the 25th DAS. This changes as of the 26th DAS, where the CO_2_ assimilation of the P deficient plants declines very fast, while it remains relatively constant for the P sufficient plants. Also in the 2nd experiment, the application of P via the foliage led to a slower decrease in the photosynthetic rate over time. The 1st foliar application of P (24 DAS) resulted in a 4 µmol CO_2_ m^−2^ s^−1^ (22%) higher CO_2_ assimilation rate over a period of 15 days compared to the P-deficient control plants. Following the 2nd foliar application (30 DAS), the difference in the assimilation rate is lower (2.97 µmol CO_2_ m^−2^ s^−1^), but the relative difference between the two variants is greater (48%) (Figure 5a).

### 2.4. SPAD Measurements

When comparing P deficient with P sufficient control plants it became evident that P supply had a significant influence on the chlorophyll content of maize plants, as derived from SPAD values. While values for the P-sufficient control became significantly higher on the 7th day of the measuring period and rose to a level of 45 to 50, the SPAD value of the P-deficient control plants decreased until reaching 16 SPAD units at the end of the experiment (Figure 5b). When looking at the plants resupplied with P via foliar application, it can be seen that in the current trials the timing of foliar application had no influence on leaf SPAD values. However, a P foliar application led to overall significantly higher SPAD values compared to the P deficient control plants. But still, with values of 28.3 (1st foliar application) and 28.8 (2nd foliar application) on the 21st day, the SPAD values of the P sufficient control (44.6) could not be reached (Figure 5b).

## 3. Discussion

Phosphorus deficiency is a genuine problem for arable farming that is likely to increase in the future, thus reducing the productivity of dryland crops such as maize [3,17]. Foliar P fertilisation provides the opportunity to improve the P supply in P deficient plants [57]. However, it remains uncertain how long the benefit of a P foliar application lasts and what immediate influence it has on physiological parameters and plant growth over time after application. This study, therefore, focussed on evaluating for the first time the temporal relationship between a P foliar application and the development of photosynthesis parameters and biomass formation over a period of 14 and 21 days using maize plants as a model crop.

In fully expanded maize leaves, the P concentration of well-nourished plants is in the range of 3.5 to 6 g kg^−1^ DM [58]. Accordingly, the control plants supplied with 0.2 mM KH_2_PO_4_ in the nutrient solution were in the P sufficiency range (data not shown), whereas the P deficient control plants, which only received 0.01 mM KH_2_PO_4,_ were clearly below this threshold (Figure 1a). Resupply of P via 200 mM KH_2_PO_4_ foliar application markedly increased tissue P concentrations as reported before (e.g., [57]). Jezek et al. [29] showed that, after foliar magnesium (Mg) application, which, like P, is very phloem-mobile [17], no tissue Mg increases could be detected in roots and younger shoots. This was not the case after P foliar application in our study, because P concentrations significantly increased in all plant components analysed (Figure 1), which is likely associated with source-sink relationships in growing plants. Leaves that are 40–60% developed are transformed from sink to source organs, and from this moment they redistribute and translocate resources to sink plant parts such as younger leaves and roots [59,60]. This is most likely the reason why the P concentration in the younger leaves (Figure 1c) and the roots (Figure 1e) of P foliar sprayed plants were elevated for ten days. This is six days longer than in the older shoot with mature leaves (Figure 1b), where the P concentration was only elevated for four days. Koontz and Biddulph [61] recorded similar results in bean plants (*Phaseolus vulgaris* L.), where ^32^P was only redistributed from fully developed leaves into growing tissues and not the opposite way. Comparable findings have been reported by various authors (e.g., [24,57,62]). However, the P concentration of the foliar P applied plants in all plant components decreased over time to the level of the P deficient control plants (Figure 1).

One factor influencing the change in P concentration was biomass formation, which was significantly higher in foliar P applied plants than in the P deficient control seedlings from day 6 after foliar P application. Thus, comparing P concentration and biomass development, it seems most likely that a dilution of P concentration took place. (Figure 2, [63]). Positive biomass effects of foliar P application were also reported by Noack et al. [30] and were clearly visible in all plant components in our experiments (Figure 2). Nevertheless, after a short phase of accelerated biomass production in the foliar P applied seedlings (day 4–10), plant development followed a similar pattern to that of P deficient plants starting from day 10 (Figure 2). It can be assumed that the growth-promoting influence of P foliar application is not systematically maintained in the further course of vegetation until maturity, as shown by Görlach et al. [28]. In our study, P deficient plants not only showed a distinctly reduced biomass production (Figure 3a) but also a lower leaf area as well as early leaf senescence compared to P sufficient plants (Figure 3b), which is in agreement with several other studies (e.g., [17,57,64]). Foliar P application was able to reduce premature leaf senescence but could not prevent it completely (Figure 3b).

The CO_2_ assimilation of P-deficient and P-sufficient plants was very similar on days 1 and 2 in the measurement period of the 2nd experiment, although P-deficiency already lasted for eight and nine days, respectively (Figure 5a; day 0, 1). In higher plants, the vacuole acts as a storage pool of P, and under adequate P supply, around 85–95% of total P is located in the vacuoles as P_i_ [17,65]. This allows the vacuole to act as a buffer to keep the cytosolic P_i_ concentration constant, which is crucial for the maintenance of photosynthetic processes [66]. However, under severe P deficiency, the cytosolic P_i_ concentration also decreases, which most probably happened in this experiment after ten days of P deficiency and explains the rapid decline of CO_2_ assimilation in the P deficient plants from day 2 in the measurement period (Figure 5a). Severe P deficiency can lead to almost complete inhibition of photosynthesis [17,67,68], which also occurred in our study, as seen in Figure 4a and Figure 5a. Phosphorus resupply to P deficient plants via foliar application (2nd experiment, day 1, 24 DAS) did not stop the decline of CO_2_ assimilation, but significantly slowed it down (Figure 5a). The same effect could be shown by a P foliar application one week later (2nd experiment, day 7; 1st experiment, day 0; 30 DAS) (Figure 4a and Figure 5a), indicating that the application timing has only a minor influence on the effect of the P foliar application within the time frame of one week. Despite the significant positive influence of foliar P application on photosynthesis, the performance of a P-sufficient maize plant could not be achieved (Figure 4a and Figure 5a), as also reported by Eichert et al. [69] after iron (Fe) spraying of Fe deficient peach leaves. This is contrary to the results of Jezek et al. [29], who showed a full recovery of CO_2_ assimilation as a result of foliar Mg application. Since the tissue concentrations of Mg (2.5–6 g kg^−1^ DM) and P (3.5–6 g kg^−1^ DM) in well-nourished maize plants [58] are meant to be similar, an insufficient amount of P provided by foliar application cannot be the only cause for the inability to restore plant functionality and consequently, there must be other reasons. In Figure 5a, an increase in CO_2_ assimilation in the P deficient and foliar P applied plants can be seen from day 6. This was caused by a nutrient solution change in which only a very small amount of P (2.8 mg P pot^−1^) was provided compared to the foliar application (93 mg P pot^−1^). Nevertheless, CO_2_ assimilation was increased by about 85%, indicating that P-deficient maize plants are not fundamentally incapable of recovering plant functionality, but that the uptake of P via the leaf is the limiting factor. In the study of Arsic et al. [53], P deficient barley plants (*Hordeum vulgare*) were also unable to restore photosynthetic processes after foliar P treatment. As suggested by the authors, one reason was the altered morphology of the plant under P deficiency, which showed no influence on stomatal or trichome density, unlike in Fernández et al. [25], but a thicker cuticle and epidermal cell wall with lower abundance of polysaccharides, all of which are characteristics that are connected to impaired foliar uptake [31,53]. Ionic radius might also be an influencing factor for the insufficient P uptake, which with a value of 223 pm for H_2_PO_4_^−^ in the dehydrated form is considerably larger than e.g., Mg^2+^ with only 65 pm [17,53]. The decline of the transpiration rate in P deficient plants was similar to the course of photosynthesis (Figure 4b) and is in line with the results of Sitko et al. [70] and Veronica et al. [71] and can be attributed to reduced leaf size and stomatal conductance, as our results confirm (data not shown, Figure 3).

Chlorophyll is generally considered a non-limiting compound, which only changes under severe malnutrition [72]. That is probably why chlorophyll contents estimated by SPAD measurements showed a delayed response to insufficient P supply compared to CO_2_ assimilation, indicating that it was not the key limiting factor for declining photosynthesis (Figure 5). However, it is likely that P is not the primary reason for the observed decrease in maize leaf SPAD values. While some studies suggest that a better P supply leads to higher chlorophyll contents ([72,73]), Peng et al. [74] have even found a slight increase in SPAD values with P deficiency in rice plants, as long as nitrogen (N) is available in sufficient amounts. In both P deficient and foliar P applied plants, anthocyanin pigments were visible as a result of P deficiency, causing a purple-blue colouration of the leaves (Figure 3b). These pigments may affect the validity of the SPAD measurement based on leaf transmittance [75], what questions the usefulness of carrying out SPAD measurements in strongly P deficient plants. However, anthocyanin pigments can be only responsible for an increase in SPAD values [75] and are hence not related to the SPAD values decline recorded in our experiments. This may likely be associated with early leaf senescence, which was strongest in the P deficient plants (Figure 3b), and can cause a major decline in chlorophyll content determined by SPAD values [75].

## 4. Material and Methods

### 4.1. Experimental Setup

Experiments were carried out under controlled conditions in the experimental station of the Institute of Plant Nutrition and Soil Science at Kiel University (54°20′50″ N, 10°6′55″ E). Maize plants (*Zea mays L*., cv. Keops, KWS SAAT SE & Co. KGaA, Einbeck, Germany) were grown in a greenhouse under a photoperiod of 15 h (7 a.m. to 10 p.m.) and at a day/night temperature of 20/15 °C. Irradiation was around 250 µmol photons m^−2^ s^−1^ and relative humidity was maintained at 50 ± 15%. Temperature and humidity were regulated by automatic air exchange. For controlling the growth conditions and nutrient supply, plants were transferred to a nutrient solution after germination in a 2 vermiculite: 1 perlite mixture. For this purpose, two nine-day-old seedlings were transferred into one black, opaque 10 L pot. In the 1st week, the maize plants received 25% and after a continuous increase from the 2nd week onwards 100% of the nutrient solution concentration. The composition of the nutrient solution was as follows: 1.3 mM Ca(NO_3_)_2_, 0.7 mM NH_4_NO_3_, 1.0 mM K_2_SO_4_, 2.0 mM CaCl_2_, 0.5 mM MgSO_4_, 200 μM Fe-EDTA, 5 μM H_3_BO_3_, 2 μM MnSO_4_, 0.5 μM ZnSO_4_, 0.3 μM CuSO_4_, and 0.01 μM (NH_4_)Mo_7_O_24_. The KH_2_PO_4_ concentration of the nutrient solution, which was primarily used as a P source, was individually adjusted depending on the variants. Nutrient solution was changed on a weekly basis, like the rearrangement of the pots, following a completely randomized design.

The 1st experiment focused on assessing the uptake of P via the leaf after foliar P fertilisation and its influence on biomass formation and plant physiology parameters over a period of time and under different P nutritional regimes. In order to initially enable good seedling development, all 72 pots were supplied with a sufficient amount of P (0.2 mM KH_2_PO_4_) during the first seven days in the nutrient solution. Subsequently, the pots were divided into three variants—(1) plants that continued to be sufficiently supplied with P throughout the whole growth period (12 replicates, P_suff_-control), (2) plants that received 0.01 mM KH_2_PO_4_ and were though P deficient for the rest of the growth period (32 replicates, P_def_-control) and (3) plants that were treated as (2) but were re-supplied with P provided as a P foliar treatment after two weeks (28 replicates, P_def_ + FA), as described in Section 2.2 (Figure S1). To determine the nutritional status of the plants prior to P foliar application and the following measuring period that begins with it, four biological replicates, each of the P deficient and P sufficient control were harvested on the day of foliar application (30 days after sowing (DAS), Figure S1). To subsequently investigate the P uptake over time, further harvests of four biological replicates each of the P deficient control and the foliar applied variants were made two, four, six, eight, ten, 12 and 14 days after the P foliar application. The harvest of the P-sufficient control plants took place on days 8 and 14 (Figure S1). To gain insight into P uptake via the leaf as well as into translocation, the harvested plants were divided into five components: (1) Shoot below 4th leaf, which corresponds to the older leaves and the stem, (2) 4th leaf, which was the youngest fully emerged leaf, (3) shoot above 4th leaf, which is equivalent to the younger leaves, (4) the youngest leaves, which were still rolled up and encased by other leaves at the time of foliar application and thus did not receive drops of the foliar P fertiliser and (5) the root. Furthermore, the temporal changes with regard to physiological parameters and chlorophyll content of the leaves were determined as described in Section 2.3.

For more detailed investigations of physiological parameters and SPAD values in different P treatments, a 2nd experiment was developed under the same conditions as the 1st experiment. A total of 16 pots were divided into four variants: P_suff_-control, P_def_-control, P_def_ + FA1, and P_def_ + FA2. The experimental design was the same as in the 1st experiment, with the difference of an additional, single P foliar application, one week earlier (24 DAS). In the P_def_ + FA2 variant, P foliar application was performed on the same day as in the 1st experiment (30 DAS). Due to the earlier, 1st foliar application, the measuring period was shifted forward by seven days and thus amounted to a total of 21 days.

### 4.2. Foliar Treatment

Before applying the foliar P fertiliser, the stem base was wrapped with absorbent paper and all potential entry points for liquids in the lid were additionally covered with aluminium foil to prevent P contamination of the nutrient solution. The foliar fertiliser solution consisted of 200 mM KH_2_PO_4_ and 0.1% (*v*/*w*) Silwet^®^ Gold (Spiess-Urania, Ochsenfurt, Germany) and was supplied to the adaxial and abaxial side of leaves and shoots using a calibrated hand-spray bottle until the surfaces were fully wetted. This implied the delivery of 12 mL (74 mg P pot^−1^) foliar spray solution per pot for the application at 24 DAS (2nd experiment) and 15 mL (93 mg P pot^−1^) per pot for the application at 30 DAS (1st and 2nd experiment). Foliar treatments were carried out in the early morning hours between 4 a.m. and 6 a.m. to benefit from high relative humidity and to avoid leaf burn due to excessive irradiation. Plants received a onetime foliar spray. Foliar treatments were carried out as previously described by Görlach et al. [56].

### 4.3. SPAD Values and Measurement of Physiological Parameters

Chlorophyll contents were non-destructively estimated with a portable chlorophyll meter (SPAD-502, Minolta, Marunouchi, Japan). Measurements started with 23 DAS in the 2nd experiment and were taken on a daily basis between 1 and 2 p.m. until the end of the measurement period (44 DAS). Thus, six measurement points distributed over the 4th leaf were considered and a mean value was calculated. For each treatment, four biological replicates were measured.

Physiological parameters such as net photosynthetic rate and net transpiration were measured using a portable, open-flow gas exchange system (LI-6400, LI-COR Biosciences, Lincoln, NE, USA). Measurements were taken from 10 a.m. to 1 p.m. on light-adapted plants on the same days and on the same plants as the SPAD measurements (Figure S1). The measurement area was 6 cm^2^ of the leaf lamina in the middle of the 4th leaf without the midrib. This area was marked so that the daily measurements were always taken in the exact same spot. Gas exchange measurements were performed at 2500 µmol m^−2^ s^−1^ photosynthetic photon flux density (PPFD) using the LED light source (LI-6400-02, LI-COR Biosciences, Lincoln, NE, USA). The flow rate was set to 500 µmol s^−1^ and the CO_2_ concentration of the incoming air was regulated to 405 µmol mol^−1^ by CO_2_ injection (LI-6400-01, LI-COR Biosciences, Lincoln, NE, USA). The temperature in the measuring cuvette was set to 25 °C.

### 4.4. Plant Tissue Sampling and Analysis

In order to remove the foliar P fertiliser potentially adhering to the sprayed surfaces, all plant parts were thoroughly washed in a three-step process with deionized water after fresh weight determination. Subsequently, all samples were dried at 65 °C, then weighed and ground to powder (Cyclotec 1093, Foss Tecator, Höganäs, Sweden). For nutrient analysis by inductively coupled plasma-mass spectrometry (ICP-MS; Agilent Technologies 7700 Series, Böblingen, Germany), 200 mg of each replicate was transferred to 10 mL 69% HNO_3_ (ROTIPU-RAN Supra for ICP, 69%) and dissolved in an 1800 W microwave-oven (MARS 6 Xpress; CEM, Matthews, MC, USA) for 45 min, as described by Jezek et al. [29].

### 4.5. Statistical Analysis

Statistical analysis of the data was performed using R statistical package software (version 4.0.2, R foundation for statistical computing, Vienna, Austria) SPSS software (version 28.0, IBM Corp., Armonk, New York, USA) and GraphPad Prism 8 (version 8.2.1, GraphPad Software, San Diego, California, USA). The analysis was based on four or three biological replicates. The effect of the treatments was tested using Student’s *t*-test or one-way ANOVA according to Duncan’s multiple range test at *p* ≤ 5%.

## 5. Conclusions

Phosphorus deficiency strongly impaired the efficiency of physiological processes of maize plants. Foliar P application increased tissue P concentrations, which enhanced biomass formation and improved physiological processes, such as leaf photosynthesis and transpiration rates. However, the uptake of P via the maize leaf was limited, so P concentration and physiological processes declined again after a few days, and it was not possible to restore plant functionality. It is concluded that further studies are needed, for investigating aspects such as the influence of plant surface and cell structure on P uptake in maize, among other factors.

## Data Availability

The data presented in this study are available on request from the corresponding authors.

## Supplementary Materials

The following supporting information can be downloaded at: https://www.mdpi.com/article/10.3390/plants11212986/s1, Figure S1: Schematic overview of the experimental setup.
