# Supplementary material for: Foliar P Application Cannot Fully Restore Photosynthetic Capacity, P Nutrient Status, and Growth of P Deficient Maize (Zea mays L.)
Source: Plants (Basel). 2022 Nov 5;11(21):2986. doi: 10.3390/plants11212986 (PMC9654361; doi:10.3390/plants11212986)
Supplement: Supplementary file 1 [file plants-11-02986-s001.zip › plants-1996053-Supplemental Material.pdf]

Supplemental Material

Title: Schematic overview of the experimental setup

Authors: Jon Niklas Henningsen · Bruno Maximilian Görlach · Victoria Fernández · Jasper Lauritz Dölger · Andreas Buhk · Karl Hermann Mühling

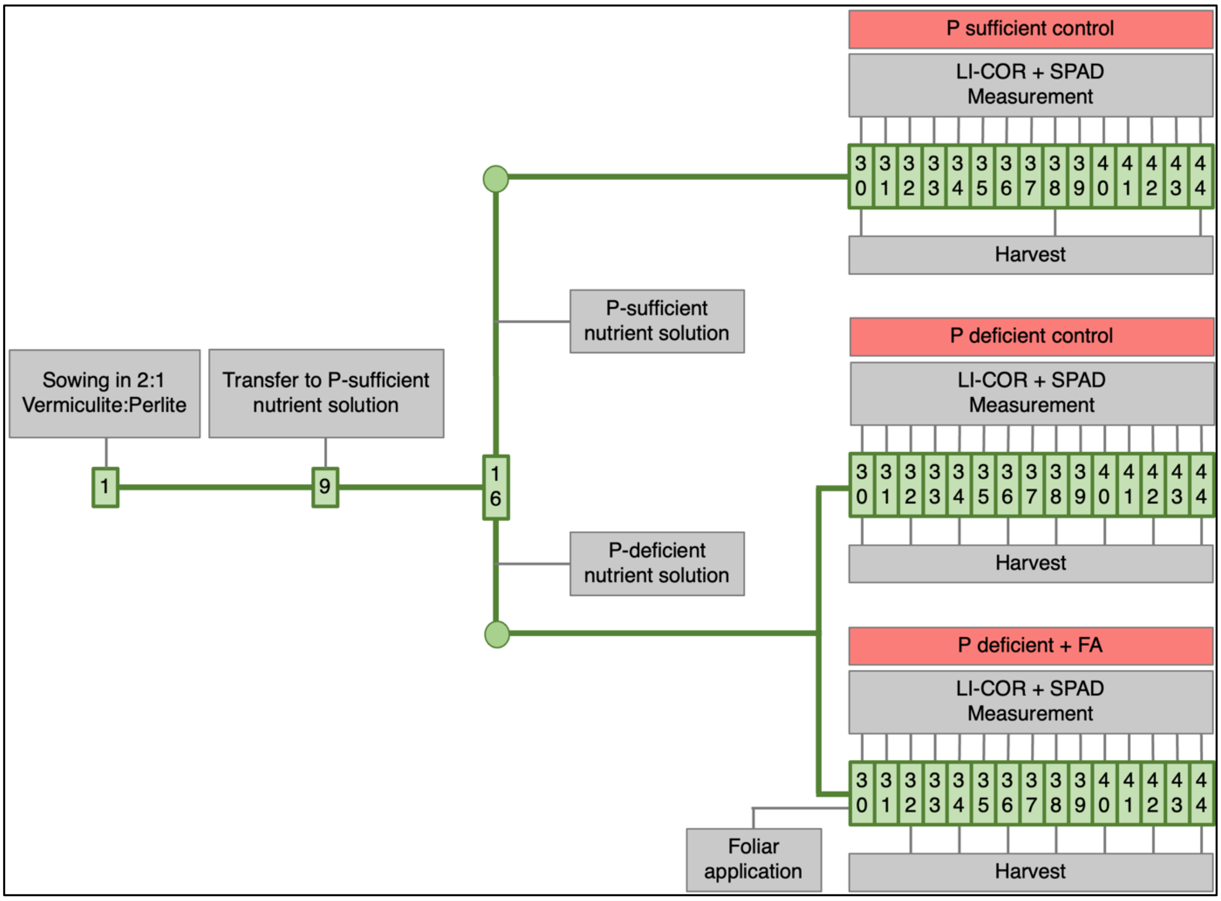

Figure S1. Schematic representation of the set-up from the 1st experiment. Multiple analytical measurements were performed on three treatments: P sufficient control, P deficient control, and P deficient + FA (foliar application). The numbers given in the green boxes indicate the days after sowing (DAS)
